# A Human Cytomegalovirus-Encoded microRNA Regulates Expression of Multiple Viral Genes Involved in Replication

**DOI:** 10.1371/journal.ppat.0030163

**Published:** 2007-11-02

**Authors:** Finn Grey, Heather Meyers, Elizabeth A White, Deborah H Spector, Jay Nelson

**Affiliations:** 1 Vaccine and Gene Therapy Institute, Oregon Health Sciences University, Portland, Oregon, United States of America; 2 Department of Pathology, Harvard Medical School, Boston, Massachusetts, United States of America; 3 Cellular and Molecular Medicine, University of California San Diego, La Jolla, California, United States of America; Duke University Medical Center, United States of America

## Abstract

Although multiple studies have documented the expression of over 70 novel virus-encoded microRNAs (miRNAs), the targets and functions of most of these regulatory RNA species are unknown. In this study a comparative bioinformatics approach was employed to identify potential human cytomegalovirus (HCMV) mRNA targets of the virus-encoded miRNA miR-UL112-1. Bioinformatics analysis of the known HCMV mRNA 3′ untranslated regions (UTRs) revealed 14 potential viral transcripts that were predicted to contain functional target sites for miR-UL112-1. The potential target sites were screened using luciferase reporters that contain the HCMV 3′UTRs in co-transfection assays with miR-UL112-1. Three of the 14 HCMV miRNA targets were validated, including the major immediate early gene encoding IE72 (UL123, IE1), UL112/113, and UL120/121. Further analysis of IE72 regulation by miR-UL112-1 with clones encoding the complete major immediate early region revealed that the IE72 3′UTR target site is necessary and sufficient to direct miR-UL112-1-specific inhibition of expression in transfected cells. In addition, miR-UL112-1 regulation is mediated through translational inhibition rather than RNA degradation. Premature expression of miR-UL112-1 during HCMV infection resulted in a significant decrease in genomic viral DNA levels, suggesting a functional role for miR-UL112-1 in regulating the expression of genes involved in viral replication. This study demonstrates the ability of a viral miRNA to regulate multiple viral genes.

## Author Summary

Our ability to understand the biology of viruses depends not only on functional analysis of genes they encoded but also on specific regulation of those genes during viral infection. In herpesviruses, viral gene regulation is highly complex and plays a significant role in determining the viral life cycle during acute, latent, or persistent infection. The discovery that many herpesviruses express small regulatory RNAs, known as microRNAs, has opened up a whole new area of research in regulation of gene expression. In this paper we demonstrate that a microRNA expressed by human cytomegalovirus is able to regulate multiple viral genes, including one gene thought to be crucial for both acute and latent stages of viral infection in the host. Expression of this microRNA results in a significant reduction in viral replication. This work therefore demonstrates that viral microRNAs can regulate multiple viral genes and can have significant effects on the replication of a virus.

## Introduction

microRNAs (miRNAs) are a species of regulatory RNA molecules that are involved in the control of a variety of cellular processes [1–3]. miRNAs are small single-stranded RNA species of approximately 20–24 bases in length and are initially expressed in the nucleus, where they form defined hairpin loop structures within longer primary transcripts [4]. The hairpin loop sequence containing the miRNA is recognized and cleaved by the RNAseIII Drosha complex [5] and transported to the cytoplasm by exportin 5 [6,7]. Additional processing by a second endonuclease, Dicer, produces a double-stranded RNA from which one strand is loaded into the RNA-induced silencing complex [8–11]. Targeting of transcripts by the miRNA RNA-induced silencing complex relies on complementarity between the miRNA and the target transcript. In cases of complete homology, the target transcript is cleaved, while partial homology can lead to RNA cleavage or translational inhibition [12–14]. The precise nucleotide requirements for functional binding of a miRNA to a target sequence are not fully understood. However, binding within the first 10 bases of a miRNA, especially within bases 2 to 7 of the miRNA known as the seed region, is considered particularly important [15–17].

miRNAs are ubiquitous among multicellular eukaryotic organisms and have been identified in species as diverse as plants and higher mammals [15]. The expression of miRNAs is also a phenomenon common to many DNA viruses [18], and bioinformatic and direct cloning approaches have led to the identification of over 70 novel miRNAs expressed in DNA viruses [19]. The majority of these viral miRNAs have been identified in the herpesvirus family, although SV40 and adenoviruses also encode their own miRNAs [20–33]. Herpesviruses belong to a large family of enveloped, double-stranded DNA viruses disseminated throughout nature that are able to maintain a persistent or latent infection during the lifetime of the host [34]. The herpesviruses are divided into three groups (alpha, beta, and gamma), and members of all three groups encode miRNAs, indicating that herpesviruses have utilized RNA interference (RNAi) throughout their evolution. miRNAs identified in α- and γ-herpesviruses are located within clusters in and around genomic regions associated with latent transcription. Three α-herpesviruses, herpes simplex virus-1 (HSV-1) and Marek disease virus-1 and 2 (MDV-1 and MDV-2), have been shown to encode miRNAs close to and within the minor latency-associated transcript, a non-coding RNA detected during latent infections of all three viruses [20,21,24,27]. Multiple miRNAs have been identified within two genomic regions of the γ-herpesvirus Epstein–Barr virus and are expressed during latent infection of transformed B cell lines [23,29,30]. In murine γ-herpesvirus-68 (MHV-68), tRNA-like transcripts previously identified as latency markers were found to encode a number of miRNAs, whereas the majority of the miRNAs expressed by Kaposi sarcoma-associated herpesvirus (KSHV) are processed from a single transcript also associated with latent gene expression [29]. In contrast to α and γ herpesviruses, miRNAs encoded by the β-herpesvirus human cytomegalovirus (HCMV) are located throughout the genome and were identified and detected during acute infection [25,26,29]. Following infection of human fibroblast cells, HCMV miRNAs accumulate as the infection progresses and in the majority of cases are expressed with early kinetics. Of the nine miRNAs encoded by HCMV, four are encoded antisense to open reading frames (ORFs), with the remaining encoded within intergenic regions, including UL36–1, which is encoded within the intron of the anti-apoptosis gene UL36 [35].

Validated target transcripts and regulatory functions for the vast majority of viral miRNAs remain unknown. Two likely scenarios for viral miRNA function include targeting of cellular gene expression to induce a more favorable environment for the virus, or the regulation of viral genes to establish precise temporal or tissue-specific regulation of viral gene expression. To date, viral miRNAs do not demonstrate complete homology to any known cellular transcript, suggesting that targeting of cellular genes is unlikely to occur through direct cleavage. Two recent reports have suggested that cellular genes can be regulated through incomplete binding of viral miRNAs to target sequences within cellular 3′UTRs. Gupta et al. identified a miRNA encoded by HSV-1, which the authors suggest protects infected cells against apoptosis by inhibiting the translation of the cellular genes, TGF-β1 and SMAD3 [27]. In addition, Samols et al. identified the cellular gene THBS-1 as a target for KSHV miRNAs [36]. Surprisingly, the authors found that all ten of the KSHV miRNAs targeted this gene, suggesting a remarkably high level of redundancy. Viral miRNAs have also been reported to regulate expression of viral genes. SV40 regulates the expression of its large T antigen via two miRNAs encoded directly antisense to the gene [33]. Expression of the miRNAs leads to cleavage of the large T antigen transcript and reduced levels of large T antigen during infection. There are, however, no reports of *trans*-regulation of viral gene expression by viral miRNAs through binding to target sequences with incomplete homology.

The aim of the current study was to identify and characterize functional targets of the HCMV-encoded miRNA miR-UL112-1. Using a bioinformatics approach in combination with luciferase assays, we identified three HCMV gene targets for miR-UL112-1, including the mRNA encoding the major immediate early (MIE) *trans*-activating protein IE72. IE72, and the additional MIE transactivator, IE86, are the most abundant transcripts expressed from the complex MIE region of the viral genome, are known to be critical for the induction of early and late gene expression, and are required for efficient viral replication [37]. These studies provide the first description to our knowledge of a viral miRNA that regulates multiple viral genes through binding to the 3′ untranslated region (UTR) of viral transcripts and inhibition of translation.

## Results

### miR-UL112-1 Negatively Regulates Expression from Vectors Containing the HCMV 3′UTRs of IE72, UL112/113, and UL120/121

**Figure 1:**
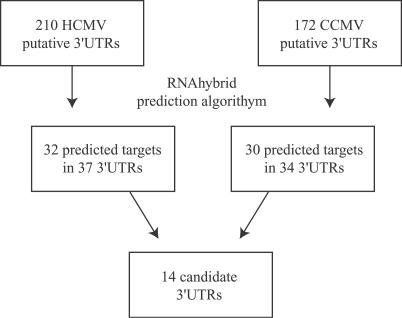
Bioinformatic Strategy for the Identification of miR-UL112-1 Targets in HCMV Genome 3′UTR sequences were determined as the 3′ end of each annotated ORF to the first AATAAA polyadenylation site. Following analysis using RNAhybrid, 14 candidate 3′UTRs contained target sites for miR-UL112-1 were identified within corresponding HCMV and CCMV 3′UTR sequences.

To determine whether miR-UL112-1 targets HCMV genes, we used a bioinformatics approach to predict potential miRNA binding sites within the 3′UTRs of HCMV transcripts. Currently, there is limited data available regarding the precise 3′ ends of HCMV transcripts. Therefore, we established a putative 3′UTR database by including sequences from the 3′ end of each annotated ORF to the first canonical AATAAA polyadenylation signal following that 3′ end. Using the online miRNA target identification algorithm RNAhybrid [38], the 3′UTR database was searched for potential miR-UL112-1 target sites. Thirty-two unique target sites for miR-UL112-1 were identified in 37 different HCMV ORFs. The total number of sites increased to 63 when we included repeated sites within overlapping 3′UTRs of neighboring ORFs. To further refine this approach, we also predicted target sites for the closely related chimpanzee cytomegalovirus (CCMV) in a parallel manner. Since miRNAs encoded by HCMV are highly conserved in CCMV and in the case of miR-UL112-1 the sequences are identical between the two viruses, we predicted that miR-UL112-1 would target the same transcripts in both HCMV and CCMV. Therefore, miRNA target sites conserved within similar 3′UTR sequences have a higher probability of being functional. Following analysis of the CCMV genome, 34 3′UTRs were predicted to contain target sites for miR-UL112-1 with 14 3′UTRs containing target sites in both HCMV and CCMV (Figure 1). A list of the 14 identified genes, along with a brief description of their functions, if known, is shown in Table S1 [39].

**Figure 2:**
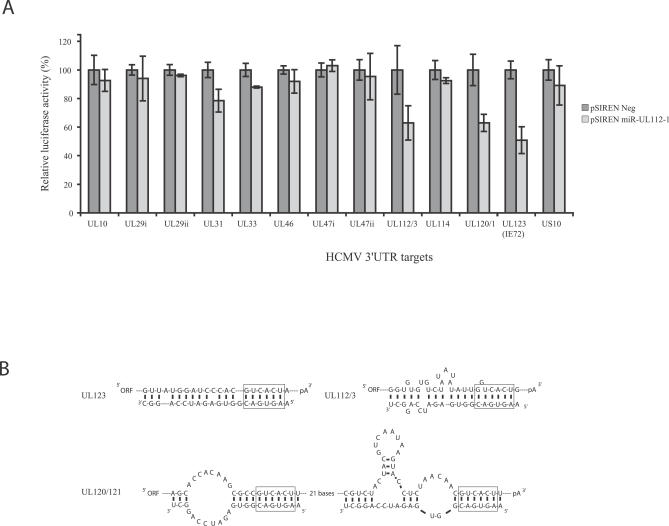
Luciferase Screening of HCMV 3′UTR Candidate Sequences (A) Luciferase screening of HCMV 3′UTR candidate sequences. Predicted target sequences were tested for their ability to inhibit expression of a luciferase reporter construct in the presence of miR-UL112-1. Either the entire 3′UTR (in the case of IE72, UL112/113 and UL120) or the predicted target sequence within 500 bases of flanking sequence were cloned downstream of the luciferase reporter gene and co-transfected with either a miR-UL112-1 expression plasmid or the same expression plasmid containing a random hairpin sequence as a negative control (pSIREN Neg). Results are shown as percentage expression of negative control sample following correction for transfection levels according to control renilla luciferase expression. Each transfection was carried out in duplicate. (B) Predicted miR-UL112-1 binding to functional target sites determined using mfold algorithm [60]. Seed region indicated by box surrounding nucleotide 2–6 of miRNA/target.

To determine whether the predicted target sequences represent functional target sites for miR-UL112-1, each of the sequences was cloned at the 3′ end of the luciferase reporter construct in pMIR (Ambion). When the 3′UTR sequence was too large to clone in its entirety, the target sequence along with 500 bases of flanking sequence was used. If neighboring 3′UTR sequences contained overlapping target sites, a single representative sequence containing the targets was used. Nucleotide coordinates of the cloned regions are listed in the Materials and Methods section. To express miR-UL112-1, the predicted coding sequence for miR-UL112-1 pre-miR was cloned into the vector pSIREN (Clontech) and expression of mature miR-UL112-1 was confirmed by northern analysis (Figure S1). 3′UTR constructs were co-transfected with either pSIREN miR-UL112-1 or the same vector expressing a randomized negative hairpin RNA supplied with the pSIREN plasmid. As shown in Figure 2, three out of the 14 luciferase constructs containing potential HCMV 3′UTR miR-UL112-1 target sequences, IE72, UL120/121, and UL112/113, directed specific inhibition of luciferase activity in the presence of miR-UL112-1. These observations demonstrate that these three HCMV 3′ UTR sequences are functional miR-UL112-1 target sites (Figure 2A). Schematic representations of predicted mir-UL112-1 binding to the Ul112/113 and UL120/121 and IE72 target sequences are shown in Figure 2B.

**Figure 3:**
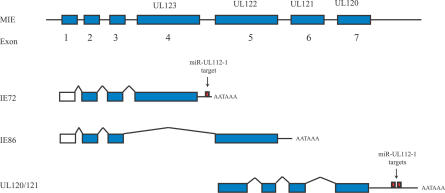
Genomic Position of miR-UL112-1 Targets Sites within the MIE Region The MIE region showing IE72 and IE86 transcripts resulting from alternative splicing are shown as well as splicing to downstream exons UL120/UL121. Position of miR-UL112-1 target sites in 3′UTR region of IE72 and UL120/UL121 indicated by red boxes.

The identification of functional target sites clustered within the 3′UTRs of the MIE region of the virus was particularly interesting. One site was identified within the 3′UTR of UL123, which encodes IE72, with two overlapping sites within the 3′UTR of ORFs UL120 and UL121 (Figure 3). Intriguingly, the site downstream of IE72 of CCMV maintained less homology to miR-UL112-1 than the equivalent site in HCMV, and would presumably form a less effective binding site. However, a second site was identified further upstream that was not found in the HCMV counterpart, suggesting that CCMV has evolved two less effective binding sites in comparison to the more homologous single site found in HCMV (Figure S2A and S2B). Interestingly, both IE72 and UL112/113 encode gene products important for viral replication, and although the function of UL120/121 is unknown, it has been suggested that UL120/121 may encode exons within the MIE family of transcripts [40].

### Expression of IE72 Protein Is Specifically Inhibited by miR-UL112-1

**Figure 4:**
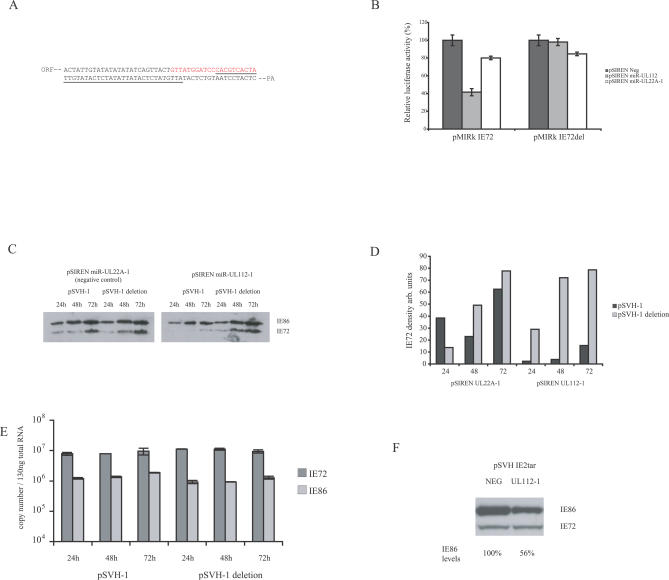
miR-UL112-1 Specifically Inhibits IE72 Protein Expression but Not mRNA Transcription (A) 3′UTR sequence of IE72 indicating predicted target sequence shown in red and the underlined region deleted in pSVH-1 deletion. (B) Luciferase construct containing either the IE72 3′UTR or the IE72 3′UTR deleted for miR-UL112-1 target sequence or luciferase construct containing the IE86 3′UTR were co-transfected with pSIREN miR-UL112-1 or the control pSIREN expressing miR-UL22A-1. Luciferase levels only inhibited in construct containing full IE72 3′UTR in the context of miR-UL112-1 expression. (C) IE72 and IE86 protein levels determined following co-transfection with genomic plasmid, pSVH-1, containing IE72 and IE86 exons and miR-UL112-1 expression plasmid or miR-UL22A-1 expression plasmid. miR-UL112-1 significantly reduces IE72 expression levels but not IE86 levels. The pSVH-1 plasmid containing deletion mutation depicted in Figure 4A was unaffected by miR-UL112-1 expression. (D) Densitometry analysis of western blot depicted in Figure 4C. (E) IE72 and IE86 RNA levels were determined by RT-PCR following co-transfection of pSVH-1 or pSVH-1 deletion with pSIREN miR-UL112-1 using primers that span intronic regions. IE72 and IE86 RNA levels were unaffected despite inhibition of IE72 protein expression in pSVH-1 construct, indicating the mechanism is post-transcriptional. Reactions that did not include RT enzyme showed no detectable signal. (F) Insertion of predicted target sequence into 3′UTR of IE86 directs miR-UL112-1-dependent inhibition, indicating the predicted sequence is functionally sufficient.

Since IE72 serves an important regulatory role during HCMV replication, we examined the regulation of this immediate early (IE) gene product by miR-UL112-1 in more detail. To confirm that miR-UL112-1-mediated inhibition of luciferase activity in the presence of the IE72 3′UTR was due to the predicted target site, a deletion mutation encompassing the 3′ half of the target (Figure 4A) was analyzed for luciferase activity. The deletion mutation completely abrogated the inhibitory effect of miR-UL112-1 (Figure 4B). Expression of the HCMV miRNA miR-UL22A-1, which is not predicted to target IE72, caused only a minor reduction in luciferase activity, and miR-UL112-1 also did not target a construct containing the 3′UTR of IE86 (unpublished data). These results indicate that miR-UL112-1 regulation of IE72 is specific and requires the predicted target sequence identified in the 3′UTR.

Following confirmation of the specific regulation of a reporter construct containing the IE72 3′UTR, we examined the effect of miR-UL112-1 on IE72 protein expression in the context of the HCMV genomic clone pSVH-1 [41] that encodes the complete IE region, including the MIE promoter driving expression of IE72 and IE86. In these experiments pSVH-1 was co-transfected with either pSIREN miR-UL112-1 or the control plasmid pSIREN UL22A-1 into 293 cells. IE86 expression provides an ideal control since expression of this IE protein is regulated by the same promoter as IE72, but the IE86 3′ UTR does not contain the miR-UL112-1 target sequence. Total cellular protein was extracted at 24, 48, and 72 h post transfection and examined by western blot analysis using a polyclonal antibody that recognizes both IE72 and IE86. As shown in Figure 4C, co-expression of miR-UL112-1 in the context of pSVH-1 significantly reduced the levels of IE72, but not IE86, expression in comparison to a vector expressing a miRNA not targeting this 3′UTR. These observations are consistent with the luciferase results, suggesting that the negative regulatory effects of miR-UL112-1 are specific to messenger RNAs containing the predicted miRNA target site. However, the level of IE72 reduction was much greater than in the luciferase reporter experiments, with a decrease in protein expression of approximately 14-fold as determined by densitometry (Figure 4D). This difference may reflect inherent variations in the assays or more effective inhibition of the endogenous IE72 viral transcript compared to the artificial luciferase reporter transcript. In addition, the inhibitory effects of miR-UL112-1 were not observed using a pSVH-1 construct deleted for the target sequence (Figure 4C and 4D). Additional smaller mutations within the target region also disrupted the inhibitory effect of miR-UL112-1, including mutations within the seed sequence (Figure S3). Interestingly, point mutations that introduced G:U base pairing within the seed region did not disrupt the inhibitory effects of miR-UL112-1, which is consistent with recent data [42] indicating that Watson and Crick base pairing within the seed region is not essential for target function (Figure S3).

Recent studies have suggested that degradation of target transcripts may occur even if the target site contains only partial homology to the miRNA. Degradation is thought to occur through the accumulation of miRNA-targeted transcripts in proximity to cytoplasmic RNA processing regions called P bodies [43–45]. To determine whether the reduction in IE72 levels following expression of miR-UL112-1 was due to translational inhibition, or the result of mRNA degradation, IE72 and IE86 transcript levels were determined by reverse transcriptase (RT)-PCR following co-transfection of pSIREN UL112–1 with pSVH-1 or the pSVH-1 deletion mutant. As seen in Figure 4E, IE72 RNA levels were unaffected by miR-UL112-1 expression, indicating that the reduction in IE72 protein levels occurs primarily through a post-transcriptional mechanism.

Finally, to determine whether the predicted IE72 target sequence was sufficient to direct inhibition of gene expression through miR-UL112-1, the predicted 21-base target sequence was introduced into the 3′UTR of IE86 in the IE72 target deletion background of pSVH-1. As shown in Figure 4F, expression of miR-UL112-1 led to a reduction in IE86 protein levels, although not to the same degree previously shown with IE72. This suggests that complete function of the target sequence may require additional flanking sequences. Thus, we have demonstrated that in the context of either a luciferase reporter construct containing the IE72 3′UTR or with a genomic expression vector containing the complete MIE region, miR-UL112-1 specifically targets the expression of IE72 through a conserved target site within the IE1 3′UTR. The target site is necessary and sufficient to direct miR-UL112-1 specific inhibition of expression, and this effect occurs through translational inhibition rather than degradation of the transcript.

### Pre-Expression of miR-UL112-1 Reduces Both IE72 Expression and Viral DNA Replication

Although the effect of miR-UL112-1 on the down regulation of IE72 expression from a genomic vector is clear, the importance of this inhibition during viral infection is unknown. We have previously shown that during the early stages of HCMV infection, when IE72 expression levels are high, miR-UL112-1 levels are low. miR-UL112-1 does not accumulate to significant levels until late in infection [26]. Furthermore, previous studies have demonstrated that disruption of IE72 expression decreases efficient viral replication at low multiplicities of infection [46,47]. Therefore, we predicted that expression of miR-UL112-1 prior to infection with HCMV would lead to a block in IE72 expression, potentially leading to disruption of efficient HCMV replication.

To determine the effect of pre-expressing miR-UL112-1 on production of IE72 as well as on viral DNA replication during infection, we used a commercially produced synthetic miR-UL112-1: a short double-stranded RNA with the exact sequence of mature endogenous miR-UL112-1. To first demonstrate that the synthetic miR-UL112-1 can inhibit IE72 expression as effectively as the pSIREN miR-UL112-1 construct, U373 cells were co-transfected with pSVH-1 and either synthetic miR-UL112-1 or a random sequence negative control pre-miR (Ambion). As shown in Figure S4, synthetic miR-UL112-1 RNA effectively inhibits IE72 expression.

**Figure 5:**
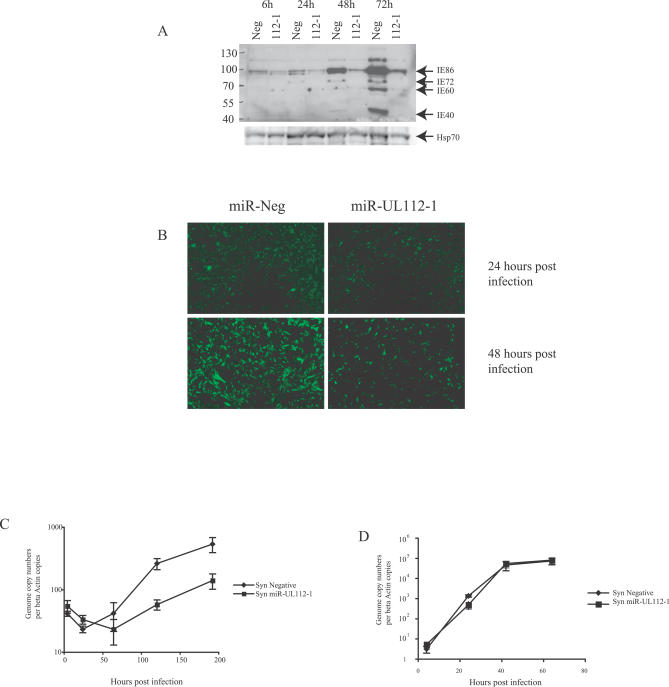
miR-UL112-1 Inhibits Immediate Early Gene Expression and Viral DNA Replication U373 cells were transfected with either synthetic miR-UL112-1 or a random sequence negative pre-miR RNA followed by infection with AD169gfp at a multiplicity of 0.5 plaque-forming units per cell. (A) Total protein was harvested and analyzed by western blot using an antibody that cross reacts with MIE genes. miR-UL112-1 transfection resulted in significant reduction in immediate early gene expression. (B) Levels of gfp were determined by microscopy at 24 and 48 h post infection, demonstrating a reduction in gfp fluorescence in cells transfected with miR-UL112-1 at later time points. (C) DNA was isolated from infected cells at the indicated time points and viral DNA levels were determined by real-time PCR and corrected against beta-actin copies. Transfection of miR-UL112-1 (syn miR-UL112-1) results in consistently lower levels of HCMV DNA at later time points compared to cells transfected with randomized control miRNA (syn negative). (D) DNA replication of HSV-1 is unaffected by miR-UL112-1 expression. U373 Cells were transfected with either miR-UL112-1 or the negative control RNA and infected with HSV at a multiplicity of 0.1. DNA levels were determined by PCR and corrected against beta-actin copies.

We next determined whether transfection of U373 with synthetic miR-UL112-1 RNA cells prior to infection with HCMV could effectively block IE72 expression. U373 cells were therefore transfected with either the synthetic miR-UL112-1 or the negative control RNA followed by infection with an AD169 strain containing an eIF1-α driven green fluorescent protein (gfp) cassette inserted into the UL21.5 gene. Cells were infected at a multiplicity of 0.5 plaque-forming units per cell. Total protein was harvested between 24 and 72 h post infection, and levels of viral immediate early proteins were determined through western blot analysis using a polyclonal antibody that recognizes IE72, IE86, and two other products of the MIE region. As seen in Figure 5A, miR-UL112-1 significantly inhibited the expression of IE72. However, additional proteins encoded by the IE region were also decreased in expression. This result is in contrast with the specific regulation of IE72 observed following transfection with pSVH-1 and does not reflect the phenotype observed for IE72 knock-out viruses. This observation is not entirely surprising, as we have previously shown that two additional viral genes other than IE72 contain functional target sites for miR-UL112-1. We also predict that other viral targets exist that were not identified through our bioinformatics screen. Expression of miR-UL112-1 is therefore likely to have pleiotropic effects on viral infection, resulting in a phenotype distinct from IE72 knock-out viruses. Targeting of IE72 by miR-UL112-1 therefore likely contributes to the phenotypic effects shown in this report, but is unlikely to be the only factor involved.

An attenuation of viral replication caused by an inhibition of IE72 expression may also contribute to a global reduction in viral gene expression due to decreased levels of viral template. Consistent with this model, the levels of gfp fluorescence expressed by the virus were significantly reduced in cells transfected with synthetic miR-UL112-1 (Figure 5B), suggesting attenuation in viral replication or infection. To confirm whether transfection of miR-UL112-1 results in a reduction in the efficiency of viral replication, viral DNA levels were analyzed by real-time PCR. Viral DNA levels were consistently lower in cells transfected with miR-UL112-1 than in control cells at later time points, indicating that the miR-UL112-1 has a significant negative effect on acute replication of the virus (Figure 5C). Importantly, transfection of cells with miR-UL112-1 had no effect on DNA replication of HSV-1, indicating that the reduction in HCMV DNA replication caused by miR-UL112-1 is not due to a general attenuation of DNA viral replication (Figure 5D). Together, these results demonstrate that pre-expression of miR-UL112-1 has pleiotropic effects on HCMV and that this regulation can inhibit viral DNA replication.

## Discussion

In this study, we demonstrate that a viral miRNA is capable of regulating multiple viral genes through partially homologous target sites within the 3′UTR regions of viral transcripts. In the case of IE72, regulation occurs post-transcriptionally and is dependent on a sequence within the 3′UTR of the mRNA. Transfection of cells with a synthetic miR-UL112-1RNA prior to infection results in a significant reduction in IE72 expression and downstream effects on the expression of other proteins encoded by the MIE region. Further potential targets include UL112/113, which encodes proteins involved in promoting viral DNA synthesis [48], and the ORFs UL120 and UL121, which are directly downstream of the IE86 ORF and may represent exons within the MIE region. The fact that these transcripts contain target sites in both HCMV and CCMV transcripts suggests that the regulation of these genes by miR-UL112-1 is evolutionarily conserved. We hypothesize that targeting of multiple viral transcripts by this miRNA would have significant effects on viral replication.

### Functional Role of IE72 in HCMV Infection

The MIE region of HCMV is known to express a number of regulatory proteins crucial for the efficient replication of the virus and coordination of viral gene expression. Although driven by the same promoter, differential splicing leads to the expression of multiple transcripts and proteins. The most abundant of these proteins are the major *trans*-activators IE72 and IE86, which are expressed from five exons within the MIE region. The first three exons are common to both transcripts, with differential splicing resulting in the fourth exon encoding IE72, and the fifth exon encoding IE86 [49–51]. This differential splicing allows each transcript to encode individual 3′UTRs, which results in specific regulation of IE72 by miR-UL112-1 and may contribute to the independent expression kinetics of IE72 and IE86. Both proteins are thought to be important in promoting early and late viral transcription, although only IE86 is essential for virus replication [41,46,47,52,53]. However, disruption of IE72 expression results in a significant attenuation of viral replication following low multiplicity infections [46]. Although the precise mechanism of action of IE72 is not fully understood, it is thought that the viral protein promotes transcription through interaction with cellular factors. Studies have shown that IE72 causes disruption of ND10 domains within the nucleus [54,55] and can directly interact with cellular basal transcription factors [56].

### Role of IE72 Regulation by miR-UL112-1 during HCMV Infection

The functional relevance of IE72 regulation by miR-UL112-1 is currently unclear, but this mechanism could affect viral replication in one of several ways. Expression of IE72 is down regulated late in infection, while IE86 expression continues at a high level. miR-UL112-1 may therefore be required to achieve regulation of IE72 translation during acute replication and may be important to establish the correct kinetic progress of viral gene expression. In support of this, a recent publication by White et al. has demonstrated that over expression of IE72 during acute replication correlates with a drop in efficient viral replication in fibroblast cells [53]. Regulation of IE72 by miR-UL112-1 may also be important for the establishment and maintenance of latent and persistent infection in vivo [37]. Although studies on HCMV latency are limited by the lack of effective tissue culture or animal models, studies on the related murine cytomegalovirus (MCMV) support a role for gene expression from the MIE region during latent or persistent infections [57]. IE1 and IE3 of MCMV are thought to be the functional homologs of IE72 and IE86, respectively. Long-term expression of MCMV IE1 is detected following establishment of a latent/persistent infection in the lungs of mice, and immune escape of the virus results in increased expression of IE3, which may represent an initial step in reactivation. Since IE72 is required for efficient replication of the virus and promotes acute replication by *trans*-activating early and late viral genes, negative regulation by miR-UL112-1 could therefore potentially contribute to establishing and maintaining a gene expression pattern appropriate for latent or persistent infection. Furthermore, γ-herpesviruses express multiple miRNAs during latent infection, suggesting a functional role during this stage of viral infection [23,29]. Experiments to determine the function role of IE72 regulation by miR-UL112-1 are in progress.

### Implications for the Function of Viral miRNAs

Given the relative complexity of gene expression in large DNA viruses such as herpesviruses, the potential to regulate multiple genes through a single miRNA is attractive. miRNAs require minimal genomic space and are not thought to induce innate or adaptive immune responses, a particularly attractive characteristic for viruses that maintain long-term infections of hosts. It is probable that additional herpesvirus miRNAs are dedicated to the regulation of viral transcripts, and it will be interesting to determine whether miRNAs encoded by other viruses target major *trans*-activating genes such as IE72. During the submission of this manuscript, a recent study demonstrated that miR-UL112-1 also targets the expression of the cellular gene MICB, a receptor for activated natural killer cells. Interestingly, this would suggest that single viral miRNAs have evolved the capacity to target both cellular and viral gene expression [58].

Finally, the possibility that viral miRNAs may have evolved as a mechanism to inhibit viral gene expression and viral replication presents a particularly attractive avenue for potential antiviral therapy strategies. Unlike many antivirals that artificially block viral processes, delivery of endogenous viral miRNAs could exploit the virus's own mechanisms to subdue replication. Not only might this approach be effective, but it could also be less prone to the problems of viral escape and resistance as the virus has evolved to maintain these mechanisms.

## Materials and Methods

### Genome sequences.

The annotated genome sequences for AD169 were downloaded from NCBI. Additional 3′UTR sequences deleted from AD169 were generated from the sequence of the HCMV clinical strain TR genome. 3′UTRs in the database consist of sequence from the 3′ end of each annotated ORF to the first canonical AATAAA polyadenylation sequence following that 3′ end.

### Target prediction.

The online target prediction algorithm RNAhybrid (http://bibiserv.techfak.uni-bielefeld.de/rnahybrid/submission.html) was used to identify potential target sites from the 3′UTR database [38]. The algorithm was run using default settings with the additional constraints of full Watson Crick base pairing through nucleotides 2 to 7. The predicted miRNA target base pairing schematics were produced using the online RNA folding program mfold (http://frontend.bioinfo.rpi.edu/applications/mfold/cgi-bin/rna-form1.cgi).

### Cells, viruses, and reagents.

Normal human dermal fibroblast (NHDF) cells (Clonetics) and human U373 cells (American Type Culture Collection) were cultured in Dulbecco's modified Eagle's medium supplemented with 10% fetal calf serum and penicillin-streptomycin-L-glutamine. HCMV strain AD169gfp was constructed using the AD169 BAC genome [59]. In brief, the cellular EF1-α promoter drives the gfp cassette and is inserted in the sense orientation into the UL21.5 gene at nucleotide position 39389.

### Plasmids.

Luciferase reporter construct pMIR was acquired from Ambion and was modified by replacing the ampicillin cassette with the kanamycin cassette from pdsRED2-N1 vector (region 1874 to 2806) into the NotI and ScaI site of pMIR. Renilla firefly control plasmid pRL was acquired from Promega. UL112/113, IE72, and IE86 3′UTR sequences were cDNA cloned from RNA harvested from HCMV-infected NHDF cells. Additional 3′UTR sequences were cloned directly from AD169 BAC DNA. Sequence coordinates as follows: UL10 19598–20548; UL29i 31018–31466; UL29ii 28993–29404; UL31 45826–46154; UL33 44399–46012; UL46 58472–58874; UL47i 68421–68734; UL47ii 64519–64903; UL112/113 162801–162914; UL114 160772–161167; UL120/121; IE72 170906–171005; IE86 169363–169275; US10 198659–199026. Genomic sequences were cloned into SpeI and HindIII sites of the pMIR multiple cloning region. The miRNA expression vector pSIREN was acquired from Clontech. miR-UL112-1 and miR-UL22A-1 were PCR cloned directly from AD169 BAC DNA, sequence coordinates miR-UL22A-1 27611–27750; miR-UL112-1 163123–163321. pSVH-1 contains the genomic region 168817–175524 of HCMV, which includes the MIE promoter and exons encoding IE1 and IE2. The construction of this plasmid has previously been described [41]. The pSVH-1 deletion mutant contains a 39-base deletion between AD169 nucleotide coordinates 170968–170912 and was constructed by site-directed mutagenesis. pSVH-1 IE2tar was constructed using site-directed mutagenesis. The predicted 21-base miR-UL112-1 target sequence from the 3′UTR of IE72 was inserted at AD169 nucleotide coordinate 169336 of pSVH-1 deletion within the 3′UTR coding sequence of IE2. The synthetic miR-UL112-1RNA and negative control were purchased from Ambion.

### Luciferase assays.

Assays were conducted in a 96-well format. pMIR constructs (0.2 μg) were co-transfected into 293T cells along with 0.2 μg miR-UL112-1 expression plasmid and 0.2 μg control renilla plasmid pRL using Lipofectamine 2000 according to the manufacturer's guidelines. Twenty-four hours post transfection, cells were harvested and luciferase levels measured using the Dual luciferase reporter assay system (Promega) according to the manufacturer's guidelines.

### Western blot analysis.

Cells were lysed in buffer containing 50 mM Tris (pH 8.0), 150 mM NaCl, 1% NP-40, and 1% sodium deoxycholate. Proteins were resolved by sodium dodecyl sulfate-polyacrylamide gel electrophoresis (SDS-PAGE) and transferred to Immobilon-P membranes (Millipore). The following antibodies were used: HCMV IE2 rabbit polyclonal antibody 638—antibody was raised against the entire IE2 protein—and horseradish peroxidase conjugated anti-rabbit (Amersham). Blots were visualized by Supersignal West Pico chemiluminescent substrate (Pierce) according to the manufacturer's protocol.

### Pre-miR transfection.

U373 cells were transfected with pre-miR RNAs using a modified version of the Oligofectamine protocol. A 20-μM stock (2.5 μl) of pre-miR was transfected into 24-well dishes of U373 cells in Optimem media according to the manufacturer's guidelines. Cells were transfected three times and allowed to recover for 2 h in complete media between transfections. Cells were infected at a multiplicity of 0.5 for 3 hs with AD169, then washed and overlayed with fresh complete media. Supernatant was removed and cells harvest in buffer containing 50 mM Tris (pH8.0), 150 mM NaCl, 1% NP-40, and 1% sodium deoxycholate and subjected to western blot analysis.

### RNA isolation and real-time RT-PCR.

RNA was isolated from cells using TRIzol Reagent (Invitrogen) according to the manufacturer's protocol. Total RNA (2 μg) was reverse transcribed using Omniscript RT (Qiagen) and 1 μM random hexamer primers. One-tenth of the RT reaction was used as a template for real-time PCR using TaqMan Universal PCR master mix (Applied Biosystems) on an ABI prism 7700 Sequence detector. Sequences of primers and dual-labeled TaqMan probes are IE86 forward primer ATGTCCTGGCAGAACTCGGT; IE86 reverse primer GCTGCAAGAGTGGGTTGTCA; IE86 probe VIC-CCAGTAGCACCGGCCCCACG-TAMRA; IE72 forward primer AGTGACCGAGGATTGCAACG; IE72 reverse primer CCTTGATTCTATGCCGCACC; IE72 probe VIC-AAAGATGTCCTGGCAGAACTCGTCAAACA-TAMRA.

### Viral DNA quantification by real-time PCR.

DNA was isolated from infected cells using the Qia-Amp DNA isolation kit (Qiagen) according to the manufacturer's recommendations. Real-time PCR was carried out using primers to the IE72 gene. Sequences of primers and dual-labeled TaqMan probe are: forward primer TTACCGAGTTCTGCCAGGACA; reverse primer CTGTTTCCAGAGTTGGCCGA; probe VIC-TCTCGTTGCAATCCTCGGTCACTTGTT-TAMRA. Reactions were carried out using TaqMan Universal PCR master mix (Applied Biosystems) on an ABI prism 7700 Sequence detector.

## Supporting Information

Figure S1 Northern Blot Analysis Showing Correct Expression of Mature miR-UL112-1 from pSIREN Expression Construct 293T cells were transfected with pSIREN miR-UL112-1 using Lipofectamine and RNA harvested 3 d post transfection. Lane 1, pSIREN miR-UL112-1; lane 2, pLXSN miR-UL112-1 (construct not used in this study); lane 3, RNA harvested from fibroblast cells infected with AD169 at an MOI of 5, 72 h post infection.
(2.4 MB AI).

Figure S2 Genomic Position and Predicted Binding between miR-UL112-1 and Predicted Target Sequences in CCMV (A) Genomic position of miR-UL112-1 targets sites within the MIE region of CCMV. The MIE region showing IE72 and IE86 transcripts resulting from alternative splicing are shown as well as splicing to downstream exons UL120/UL121. Position of miR-UL112-1 target sites in 3′UTR region of IE72 and UL120/UL121 is indicated by red boxes. (B) Predicted miR-UL112-1 binding to candidate CCMV target sites. Seed region indicated by box surrounding nucleotide 2–6 of miRNA/target.
(200 KB AI).

Figure S3 Mutation of IE72 Target Site Effects miR-UL112-1-Mediated Inhibition of IE72 A panel of mutations of the IE72 target site were constructed in the context of pSVH-1 and tested for miR-UL112-1-mediated inhibition. Panel of mutations is shown in Table S2. pSHV-1 constructs were co-transfected with pSIREN UL112–1 or the pSIREN negative control and IE86 and IE72 levels determined by western analysis. IE72 levels shown as percentage of negative control following densitometry analysis. Introduction of G:U base pairing within seed region did not fully disrupt miR-UL112-1 inhibition of IE72 expression. Additional mutations shown in Table S2 disrupted miR-UL112-1 inhibition of IE72 expression.
(189 KB AI).

Figure S4 IE72 Expression from pSVH-1 Is Efficiently Inhibited by pre-miR-UL112-1 pSVH-1 was co-transfected into U373 cells with either the synthetic miR-UL112-1RNA or the random negative control pre-miR. Synthetic miR-UL112-1 efficiently inhibits IE72 expression following co-transfection with pSVH-1.
(234 KB AI).

Table S1 HCMV Transcripts Predicted to Contain Target Sequences for miR-UL112-1 Target sequences were predicted using RNAhybrid and contain targets in both HCMV and the corresponding CCMV 3′UTR sequence.
(46 KB DOC)

Table S2 Mutations of the miR-UL112-1 Target Site in the 3′UTR of IE72 Seed sequence of wild-type target indicated in italics. Base substitutions shown in bold.
(26 KB DOC)

### Accession Numbers

The National Center for Biotechnology Information (http://www.ncbi.nlm.nih.gov/) annotated genome sequences discussed in this paper are AD169 (X17403) and CCMV (NC_003521). Additional 3′UTR sequences deleted from AD169 were generated from the sequence of the HCMV clinical strain TR genome (AC146906).

## Abbreviations

CCMV: chimpanzee cytomegalovirus
gfp: green fluorescent protein
HCMV: human cytomegalovirus
KSHV: Kaposi sarcoma-associated herpesvirus
MCMV: murine cytomegalovirus
MIE: major immediate early
miRNA: microRNA
ORF: open reading frame
RNAi: RNA interference
RT: reverse transcriptase
UTR: untranslated region
