# Supplementary material for: A Human Cytomegalovirus-Encoded microRNA Regulates Expression of Multiple Viral Genes Involved in Replication
Source: PLoS Pathog. 2007 Nov 2;3(11):e163. doi: 10.1371/journal.ppat.0030163 (PMC2048532; doi:10.1371/journal.ppat.0030163)
Supplement: Supplementary file 1 [file ppat.0030163.sg001.pdf]

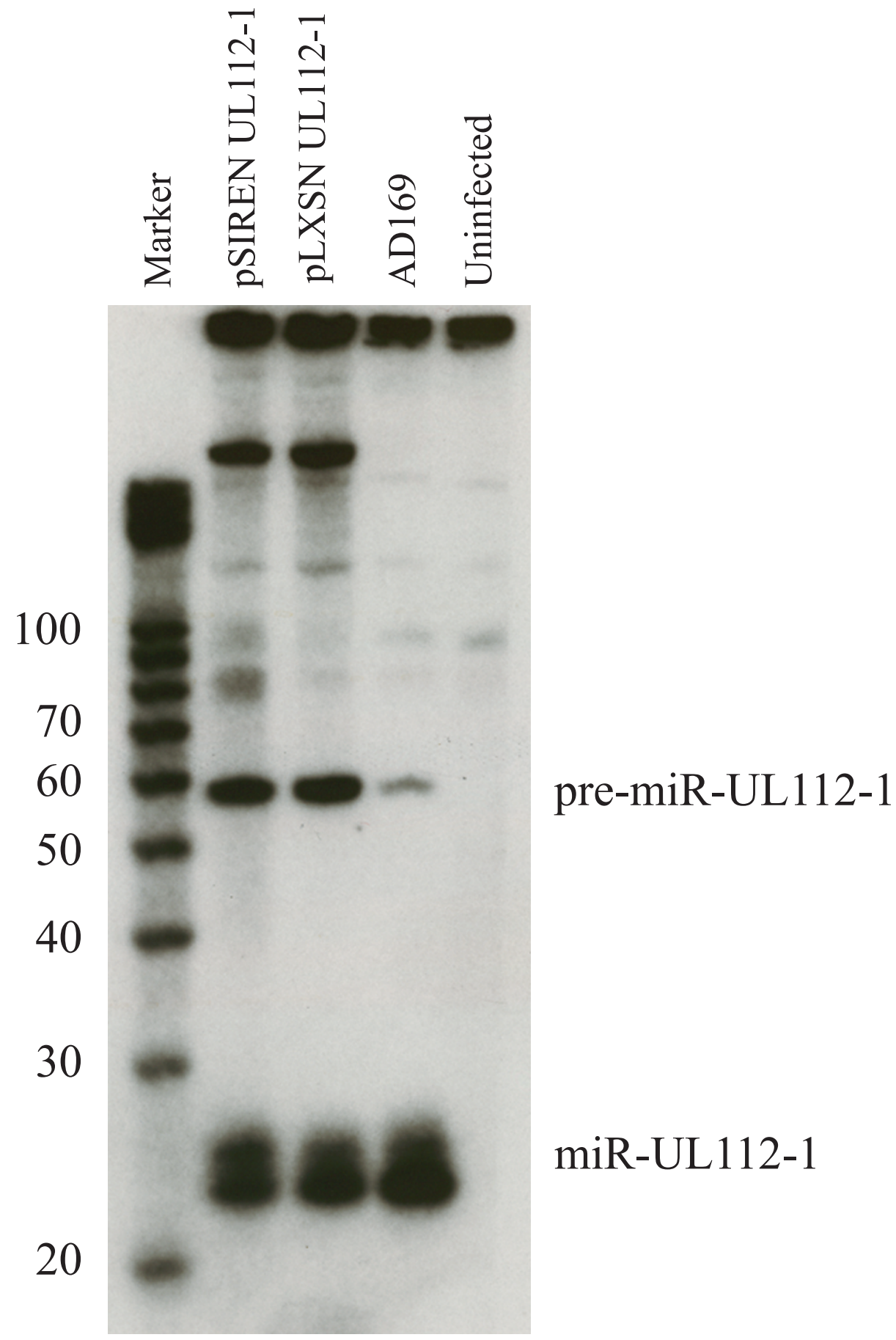

Marker
pSIREN UL112-1
pLXSN UL112-1
AD169
Uninfected
100
70
60
50
40
30
20
pre-miR-UL112-1
miR-UL112-1
