# Supplementary material for: A Human Cytomegalovirus-Encoded microRNA Regulates Expression of Multiple Viral Genes Involved in Replication
Source: PLoS Pathog. 2007 Nov 2;3(11):e163. doi: 10.1371/journal.ppat.0030163 (PMC2048532; doi:10.1371/journal.ppat.0030163)
Supplement: Supplementary file 2 [file ppat.0030163.sg002.pdf]

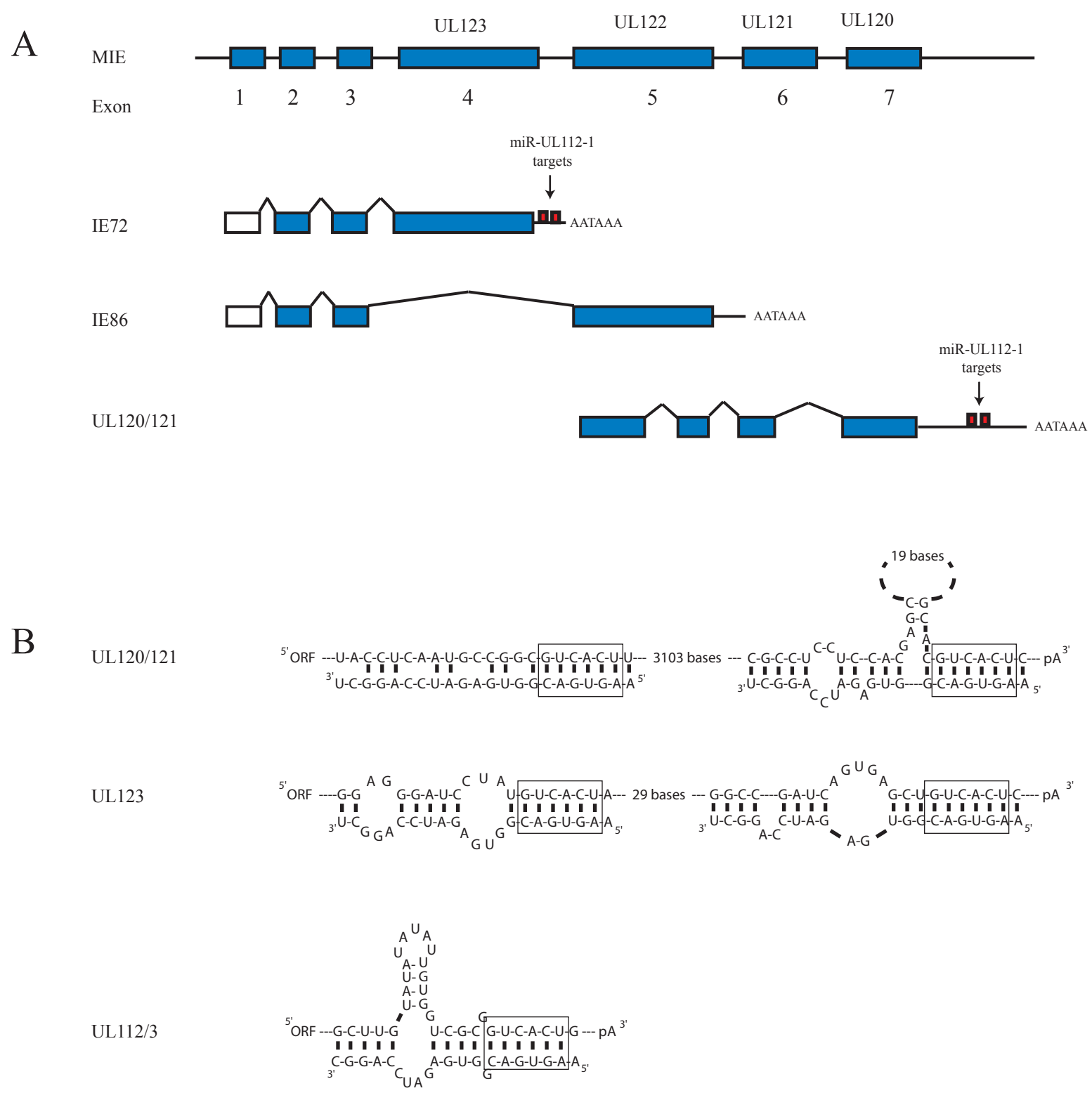

A
MIE
UL123
UL122
UL121
UL120
Exon
1
2
3
4
5
6
7
miR-UL112-1 targets
IE72
AATAAA
IE86
AATAAA
miR-UL112-1 targets
UL120/121
AATAAA
B
UL120/121
3103 bases
19 bases
UL123
29 bases
UL112/3
