# Supplementary material for: A Human Cytomegalovirus-Encoded microRNA Regulates Expression of Multiple Viral Genes Involved in Replication
Source: PLoS Pathog. 2007 Nov 2;3(11):e163. doi: 10.1371/journal.ppat.0030163 (PMC2048532; doi:10.1371/journal.ppat.0030163)
Supplement: Supplementary file 3 [file ppat.0030163.sg003.pdf]

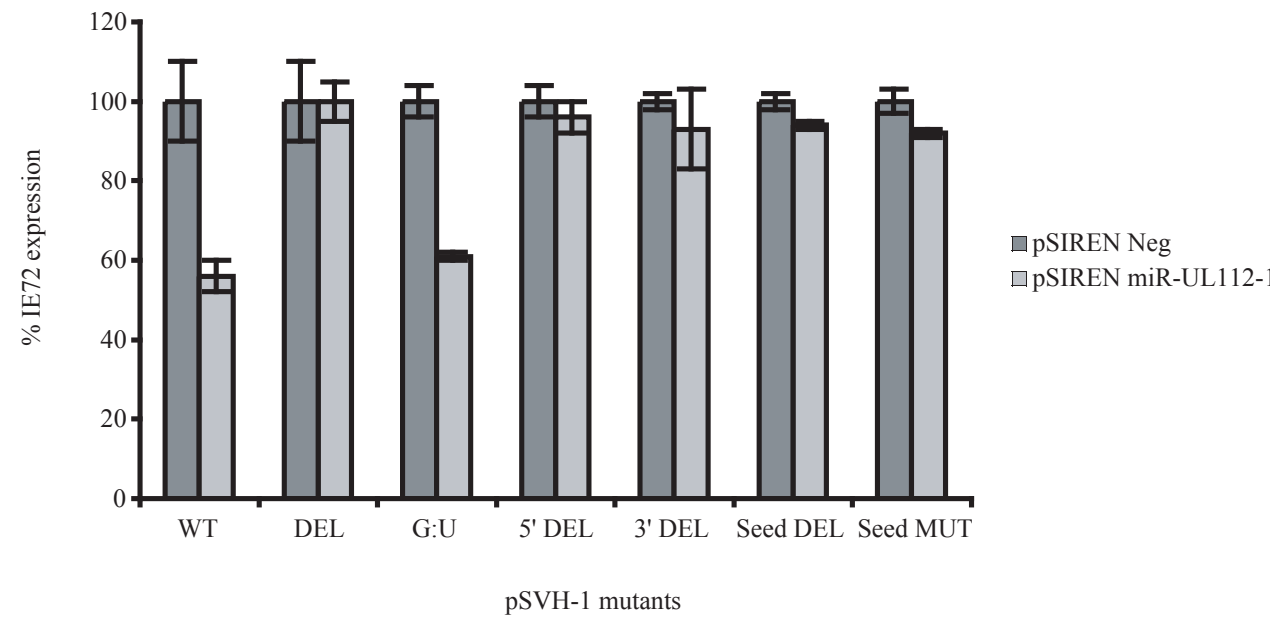
120
100
80
60
40
20
0
% IE72 expression
WT
DEL
G:U
5' DEL
3' DEL
Seed DEL
Seed MUT
pSVH-1 mutants
pSIREN Neg
pSIREN miR-UL112-1
