# Supplementary material for: A Human Cytomegalovirus-Encoded microRNA Regulates Expression of Multiple Viral Genes Involved in Replication
Source: PLoS Pathog. 2007 Nov 2;3(11):e163. doi: 10.1371/journal.ppat.0030163 (PMC2048532; doi:10.1371/journal.ppat.0030163)
Supplement: Supplementary file 5 [file ppat.0030163.st001.doc]

| **HCMV** | **HCMV Gene Family/Characteristics** | **Function/Comments** | Number of  **targets** | **3’UTR**  **length** |
| --- | --- | --- | --- | --- |
| UL9 | RL11 family; putative membrane glycoprotein | temperance for fibroblasts | 1 | 619 |
| UL10 | RL11 family; putative membrane glycoprotein | temperance for RPE cells | 1 | 2603 |
| *UL29* | *US22 family* | *temperance in RPE cells* | *3* | *8108* |
| UL30 |  |  | 3 | 9320 |
| *UL31* |  | *[HHV-7 IE gene]* | *2* | *6262* |
| *UL33x1*  *UL33x2* | *GPCR-7TM family* | *constitutive signaling, envelope protein [HHV-6A, HHV-6B, HHV-7 are chemokine receptors]* | *1* | *1605* |
| UL46 | *(core)* | *Component of capsid triplexes (minor capsid binding protein; TRI1)* | *1* | *6354* |
| *UL47* | *(core) Tegumen;* | *intracellular capsid transport; binds to UL48 protein? (LTPbp)* | *1* | *5444* |
| ***UL112*** ***UL113*** |  | *transcriptional activation, orchestration of DNA replication* | *1* | *108* |
| *UL114* | *(core)* | *Uracil-DNA glycosylase; roles in excision of uracil from DNA and temporal regulation of DNA replication (UNG) [HHV-6 IE gene]* | *1* | *3087* |
| UL120 | UL120 family; putative membrane glycoprotein |  | 2 | 534 |
| UL121 | UL120 family; putative membrane glycoprotein |  | 2 | 1190 |
| *UL123* |  | *Major immediate early protein (IE1); enhances activation by IE2; indirect effect on transcription machinery; disrupts ND10 [roseolovirus IE gene]* | *1* | *94* |
| US10 | US6 family; membrane glycoprotein | delays trafficking of MHC-I | 1 | 2704 |

**Table S1.** HCMV transcripts predicted to contain target sequences for miR-UL112-1. Target sequences were predicted using RNAhybrid and contain targets in both HCMV and the corresponding CCMV 3′UTR sequence.
