# Supplementary material for: A Human Cytomegalovirus-Encoded microRNA Regulates Expression of Multiple Viral Genes Involved in Replication
Source: PLoS Pathog. 2007 Nov 2;3(11):e163. doi: 10.1371/journal.ppat.0030163 (PMC2048532; doi:10.1371/journal.ppat.0030163)
Supplement: Supplementary file 6 [file ppat.0030163.st002.doc]

| Type of mutation | Resulting sequence |
| --- | --- |
| Wild type sequence | GTTATGGATCCCA*CGTCACT*A |
| Complete deletion | --------------------- |
| 3’ deletion | GTTATGGATC----------- |
| 5’ deletion | -----------CACGTCACTA |
| Seed sequence deletion | GTTATGGATCCCA-------A |
| G:U replacement | GTTATGGATCCC**G**CGTC**G**CTA |
| Seed sequence mutation | GTTATGGATCCCA**GCAGCGA**A |

**Table S2.** Mutations of the miR-UL112-1 target site in the 3′UTR of IE72. Seed sequence of wild type target indicated in italics. Base substitutions shown in bold.
